# Genomic landscape of alpha-variant of SARS-CoV-2 circulated in Pakistan

**DOI:** 10.1371/journal.pone.0276171

**Published:** 2022-12-13

**Authors:** Nazia Fiaz, Imran Zahoor, Saima Saima, Atia Basheer

**Affiliations:** 1 Genetic and Genomic Laboratory, Department of Animal Breeding and Genetics, University of Veterinary and Animal Sciences, Lahore, Pakistan; 2 Department of Animal Nutrition, University of Veterinary and Animal Sciences, Lahore, Pakistan; University of Bari: Universita degli Studi di Bari Aldo Moro, ITALY

## Abstract

In this study, we investigated the genomic variability of alpha-VOC of SARS-CoV-2 in Pakistan, in context of the global population of this variant. A set of 461 whole-genome sequences of Pakistani samples of alpha-variant, retrieved from GISAID, were aligned in MAFFT and used as an input to the Coronapp web-application. Phylogenetic tree was constructed through maximum-likelihood method by downloading the 100 whole-genome sequences of alpha-variant for each of the 12 countries having the largest number of Pakistani diasporas. We detected 1725 mutations, which were further categorized into 899 missense mutations, 654 silent mutations, 52 mutations in non-coding regions, 25 in-frame deletions, 01 in-frame insertion, 51 frameshift deletions, 21 frameshift insertions, 21 stop-gained variants, and 1 stop-gained deletion. We found NSP3 and Spike as the most variable proteins with 355 and 233 mutations respectively. However, some characteristic mutations like Δ144(S), G204R(N), and T1001I, I2230T, del3675–3677(ORF1ab) were missing in the Pakistani population of alpha-variant. Likewise, R1518K(NSP3), P83L(NSP9), and A52V, H164Y(NSP13) were found for the first time in this study. Interestingly, Y145 deletion(S) had 99% prevalence in Pakistan but globally it was just 4.2% prevalent. Likewise, R68S substitution (ORF3a), F120 frameshift deletion, L120 insertion, L118V substitution (ORF8), and N280Y(NSP2) had 20.4%, 14.3%, 14.8%, 9.1%, 13.9% prevalence locally but globally they were just 0.1%, 0.2%, 0.04%, 1.5%, and 2.4% prevalent respectively. The phylogeny analysis revealed that majority of Pakistani samples were grouped together in the same clusters with Italian, and Spanish samples suggesting the transmission of alpha-variant to Pakistan from these western European countries.

## Introduction

The alpha variant, also known as VOC 202012/01 or UK-variant or B.1.1.7 variant, of severe acute respiratory syndrome coronavirus-2 (SARS-CoV-2) was identified in England in November 2020 which soon became dominant in many parts of the world. This variant is speculated to be originated from some chronically infected individuals. However, due to (40–90%) increased risk of transmissibility [1, 2], high risks of infectivity and hospitalizations [3], this variant was declared as a variant of concern (VOC) on December 18, 2020 and finally in May 2021 it was named as alpha-VOC by WHO. The spread of this variant caused a second wave of pandemic in the winter of 2020 in UK and Europe with millions of new infections and subsequently imposition of a second lockdown in January 2021 [4, 5]. This variant is reported to have 14 non-synonymous, 6 synonymous and 3 deletions mutations [6]. However, N501Y mutation in the receptor-binding domain (RBD) is one of the key mutations which replace the amino acid asparagine (N) with tyrosine (Y) [7]. This mutation is considered to be involved in increasing the transmissibility of this variant by increasing the binding ability of the RBD of Spike (S) protein to human angiotensin-converting enzyme 2 (ACE2) receptors, and mediate viral entry into the host cells [8, 9]. Among the other key mutations, the deletion of amino acid at 69 and 70 positions (Δ69–70) in the S protein, had been known to be involved in potential escape of the virus from human immune response [10]. Moreover, this mutation had also been reported to have false-negative results/signals by some S-gene-targeted RT-PCR diagnostic tests [11]. Spike mutations have been reported to be involved in reducing the susceptibility of SARS-CoV-2 variants and conferring escape to the neutralizing antibodies in various in-vitro scans [12] and in some clinical isolates [13]. The N439K and Y453F mutations located in the RBD region were found to increase the binding affinity of Spike protein to ACE2, and had been reported to evade the neutralizing effects of some monoclonal antibodies (mAbs). Likewise, E484K mutation in the S protein can also help escape the virus from neutralization by polyclonal and monoclonal antibodies produced by infection or vaccination which is further augmented by the occurrence of K417N and N501Y mutations [14]. The spread of alpha-variant to almost all the countries, and occurrence of severe disease in most of the infected patients reflect its high transmissibility, and virulence. Due to these properties, the European Centre for Disease Prevention and Control (ECDC) reported that the risk associated with the introduction and community spread of this variant is very high [15]. In Pakistan, the first case of this variant was reported in last week of December 2021. And the third wave of SARS-CoV-2, which proved worst in the country with 335,728 infections and 7,849 deaths (www.covid.gov.pk), had a genomic incidence of 72.7% for the alpha-variant [16]. According to RT-PCR based diagnostic assay, the incidence of B.1.1.7 in Lahore -the 2^nd^ largest city of Pakistan- during the April 2021 (peak month of 3^rd^ wave) was 97.9%. Moreover, out of eight samples of SARS-CoV-2 which were sequenced in the start of the 3^rd^ wave of COVID-19 in Pakistan, 7 were found identical with the genomes reported from UK and one with that of Switzerland, suggesting the transmission of disease in Pakistan from these European countries [17].

In this study, we investigated the genomic variability of alpha-VOC of SARS-CoV-2 in Pakistan, in the context of global viral population of this variant. Here, we present for the very first time the most variable proteins in the SARS-CoV-2 genome, as well as the most frequent mutations in the Pakistan which also showed high dominance in the rest of the world. In this study by tracing the genome of alpha-VOC, since its introduction to Pakistan, we have detected some novel mutations which are unique to Pakistan and, likewise, some characteristic mutations of this variant which were missing in the population of alpha-VOC circulated in Pakistan. This study provides deep insight about the difference in numbers and prevalence of the mutations into the Pakistani population of alpha-variant compared with its global population which would not only help track the routes of transmission but also to develop sequence-based diagnostics, and other biologicals for the prevention and treatment of COVID-19.

## Materials and methods

The current study is mainly based on following steps: data retrieval, preprocessing and multiple sequence alignment, sequence variation analysis, and phylogenetic tree construction. We also constructed a global phylogenetic tree of alpha-VOC of SARS-CoV-2.

### Data retrieval and preprocessing, and multiple sequence alignment

As a first step, a set of 461 complete whole-genome sequences of alpha-VOC of SARS-CoV-2 samples submitted from Pakistan, were obtained from GISAID on 04 February 2022. The dataset contained genomic sequences with unique identifiers, collection & submission date, and submitting lab information, including the Wuhan-Hu-1 as a reference sequence (accession ID NC_045512.2). The data were processed and sequenced with >1% NNNs were removed from the input file. The sequence alignment of those Pakistani samples was performed using L-INS-I alignment method implemented in MAFFT (v7.480), by setting data type as nucleic acids with gap extended penalty of 0.123 and opening penalties default settings of 1.53 [16]. The Wuhan-Hu-1 sequence was used a reference genome while aligning the sequence data.

### Sequence variation analysis

For the identification of mutations, the aligned and filtered sequence file was trimmed to remove gaps compared with the Wuhan-Hu-1 reference (NC_045512.2) and used as an input to the Coronapp web application to obtain nucleotide variations [18]. Then, the genomes were clustered according to the GISAID (https://www.gisaid.org/) nomenclature by using the trimmed alignment.

### Phylogenetic tree construction

For the construction of phylogenetic tree, 100 whole-genome sequences of alpha-variant of SARS-COV-2 were retrieved from GISAID database for each of the 13 countries where most of the Pakistani diaspora is settled and used to travel to Pakistan frequently. Those countries included Australia, Canada, France, Germany, Italy, Oman, Saudi Arabia, South Africa, Spain, UAE, UK, USA, and Pakistan. The sequences were combined through MEGA-X software and aligned using MAFFT (v.7.480) [19], through multiple sequence alignment method, and manually edited by trimming the 5’ and 3’ untranslated regions and removing any gap only sites. The Wuhan/Hu-1/2019 (NC_045512.2), sampled on December 31, 2019 from Wuhan, China, was downloaded from the GISAID and used as reference genome. Finally, the phylogenetic tree was inferred by using the maximum-likelihood method based on nucleotide substitution model of Tamura-Nei (TN) model in Mega-X software [20]. Initial phylogenetic tree for the heuristic search was obtained automatically by applying Nearest-Neighbour Interchange (NNI) and BioNJ algorithms to a matrix of pairwise distances estimated using the TN model, and then, finally, maximum-likelihood phylogenetic was made by selecting the topology with superior log-likelihood value.

## Results

**Fig 1:**
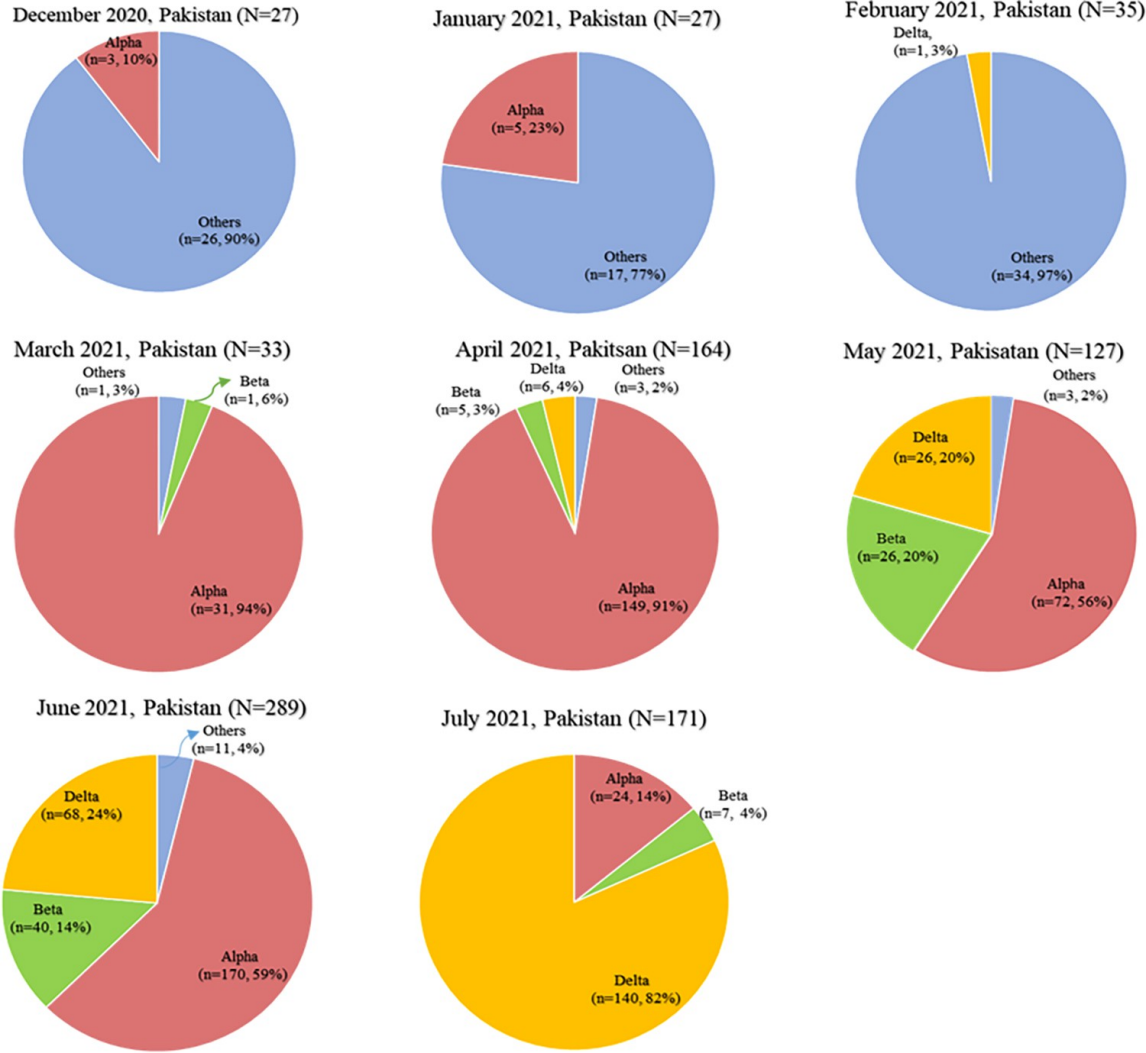
Percentages of SARS-CoV-2 genomes sequenced in Pakistan (December 2020 to July 2021).

**Fig 2:**
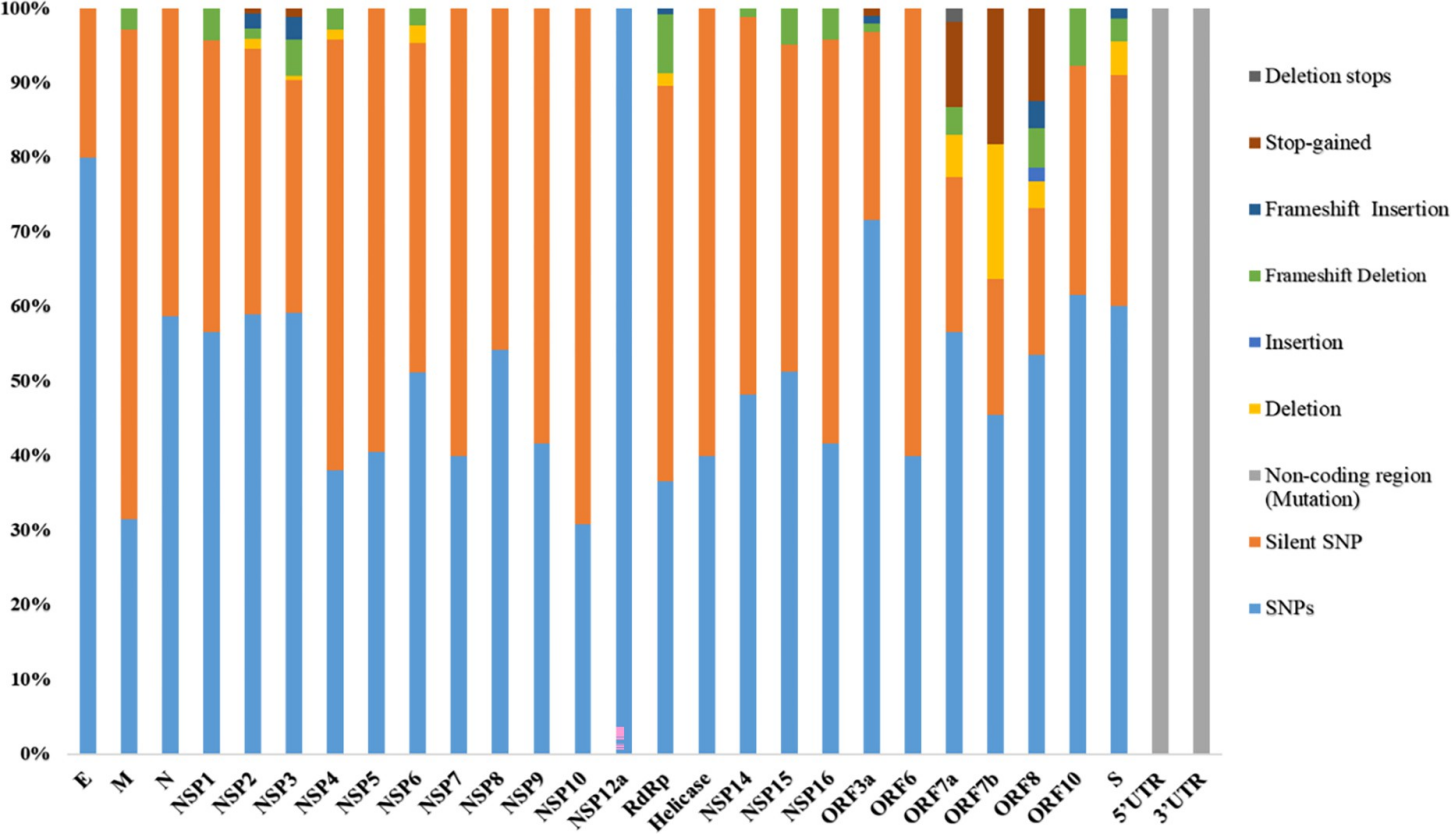
Stacked bar-chart of mutations percentages observed in different proteins of the alpha-variant population of Pakistan.

In Pakistan, the first genome of B.1.1.7 also known as VoC-202012/01 (Table 1) was sequenced in the third week of December 2020 (Table 2) and up till 4 February 2022, 461 cases of this variant had been sequenced and submitted to GISAID, which were analyzed in the current study. These sequences were from the cases of alpha-variant reported in the 2^nd^ (end of October 2020 to mid-February 2021) and 3^rd^ (mid of March 2021 to end of June 2021) wave of COVID-19 in Pakistan. The detail of alpha variant cases from December 2020 to July 2021 is presented in Fig 1. In total, we detected 1725 mutations which were further categorized as 899 amino acids changing mutations, 654 silent mutations, 52 mutations in non-coding regions, 25 in-frame deletions, 51 frameshift deletions, 01 in-frame insertions, 21 frameshift insertions, 21 stop-gained variants, and 1 deletion-stop variant (Table 3 and Fig 2).

**Table 1:** Classification of B.1.1.7 variants of concern.

Lineage	WHO	PHE	Nextstrain	GISAID
B.1.1.7	Alpha-VOC	VOC-20DEC-01, VOC-202012/01	20I/501Y.V1, 20B/501Y.V1	GRY, GR/501Y.V1

**Table 2:** Date of observation of first case of alpha-variant and number of genomes sequenced globally and, in Pakistan.

Variants	Country of origin	First case identified/Sequenced in Pakistan	First case identified /sequenced globally	Sequence counts from Pakistan	Sequence counts globally
B.1.1.7	United Kingdom	25 Dec 2020 03 Jan 2021	07 Dec 2020	461	1138400

**Table 3:** Details of mutations found in different proteins of alpha-variant of SARS-CoV-2 sampled from Pakistan.

Protein	Missense	Silent SNP	Non-coding region		In-frame		Frameshift		Stop-gained	Deletion stops	Total
			Mutation	Deletion	Deletion	Insertion	Deletion	Insertion			
**E**	8	2	-	-	-	-	-	-	-	-	10
**M**	11	23	-	-	-	-	1	-	-	-	35
**N**	64	45	-	-	-	-	-	-	-	-	109
**NSP1**	13	9	-	-	-	-	1	-	-	-	23
**NSP2**	86	52	-	-	2		2	3	1	-	146
**NSP3**	210	111	-	-	2	-	17	11	4	-	355
**NSP4**	27	41	-	-	1	-	2	-	-	-	71
**NSP5**	15	22	-	-	-	-	-	-	-	-	37
**NSP6**	22	19	-	-	1	-	1	-	-	-	43
**NSP7**	6	9	-	-	-	-	-	-	-	-	15
**NSP8**	13	11	-	-	-	-	-	-	-	-	24
**NSP9**	5	7	-	-	-	-	-	-	-	-	12
**NSP10**	4	9	-	-	-	-	-	-	-	-	13
**NSP12a**	1	-	-	-	-	-	-	-	-	-	1
**RdRp**	42	61	-	-	2	-	9	1	-	-	115
**Helicase**	22	33	-	-	-	-	-	-	-	-	55
**NSP14**	40	42	-	-	-	-	1	-	-	-	83
**NSP15**	21	18	-	-	-	-	2	-	-	-	41
**NSP16**	10	13	-	-	-	-	1	-	-	-	24
**ORF3a**	68	24	-	-	-	-	1	1	1	-	95
**ORF6**	4	6	-	-	-	-	-	-	-	-	10
**ORF7a**	30	11	-	-	3	-	2	-	6	1	53
**ORF7b**	5	2	-	-	2	-	-	-	2	-	11
**ORF8**	30	11	-	-	2	1	3	2	7	-	56
**ORF10**	8	4	-	-	-	-	1	-	-	-	13
**S**	134	69	-	-	10	-	7	3	-	-	223
**5’UTR**	-	-	13	-	-	-	-	-	-	-	13
**3’UTR**	-	-	39	-	-	-	-	-	-	-	39
**Total**	**899**	**654**	**52**	**0**	**25**	**1**	**51**	**21**	**21**	**1**	**1725**

Out of these 1725 mutations, 1058 were present in ORF1ab which transcribe into 16 non-structural proteins (NSPs). Among the ORF1ab proteins, NSP3 had the largest number of missense (210), silent (111), in-frame deletions (2), frameshift deletions (17), frameshift insertion (11), and stop-gain mutations (4). It was followed by the NSP2 which had the second highest number of mutations with 86 missense, 52 silent, 02 in-frame deletions, 02 frameshift deletions, 03 frameshift insertions, and 1 stop-gain mutation.

However, among all the proteins of SARS-CoV-2, the Spike protein had the second highest number of mutations (134 missense mutations, 69 silent SNPs, 10 in-frame deletions, 07 frameshift deletions, and 03 frameshift insertion).

Some mutations including H69, A570D, D614G, P681H, T716I, S982A, D1118H in Spike protein; P314L in NSP12b protein; T183I, A890D in NSP3; D3L, RG203KR, S235F in N; Q27*, Y73C, R521 in ORF8 had a prevalence of 95–100% in our samples, and likewise these mutations also had >95% prevalence globally (Table 4). However, some other mutations like Δ144, G204R(N), T1001I, I2230T, del3675–3677 which are also known as characteristic mutations of this variant were not present in the Pakistani population of alpha-VOC. Some new missense mutations including A52V, H164Y (NSP13), R1518K (NSP3), and P83L (NSP9) were found for the very first time in our population of alpha-VOC of SARS-CoV-2 and they were not reported earlier (Table 4).

**Table 4:** Major mutations found in the genome of alpha-variant of SARS-CoV-2 sampled from Pakistan (frequency of mutations ≥0.02).

Genomic Change	Protein	Amino acid change	Type of mutation	Mutation %	Global %	
24506T>G	S	S982A	Missense	100.0	98.8	
16176T>C	RdRp	T903T	Silent	100.0		
23271C>A	S	A570D	Missense	99.8	99.5	
23604C>A	S	P681H	Missense	99.8	99.3	
23403A>G	S	D614G	Missense	99.6	99.6	
23709C>T	S	T716I	Missense	99.6	98.9	
913C>T	NSP2	S36S	Silent	99.6		
14408C>T	RdRp	P314L	Missense	99.6	99	
14676C>T	RdRp	P403P	Silent	99.6		
24914G>C	S	D1118H	Missense	99.1	98.8	
5388C>A	NSP3	A890D	Missense	99.1	99.2	
28280GAT>CTA	N	D3L	Missense	99.1	98	
27972C>T	ORF8	Q27*	Stop-gained	98.9	99.07	
3267C>T	NSP3	T183I	Missense	98.9	99.2	
28881GGG>AAC	N	RG203KR	Missense	98.9	97.9	
28977C>T	N	S235F	Missense	98.9	98.7	
3037C>T	NSP3	F106F	Silent	98.7		
5986C>T	NSP3	F1089F	Silent	98.7		
15279C>T	RdRp	H604H	Silent	98.5		
28111A>G	ORF8	Y73C	Missense	97.6	98.8	
28048G>T	ORF8	R521	Missense	97.2	98.5	
21765TACATG>.	S	H69	Deletion	96.3	95	
21993ATT>.	S	Y145	Deletion	95.7	4.2	
6954T>C	NSP3	I1412T	Missense	95.2	99	
241C>T	5’UTR	241	Extragenic	78.3		
28273A>.	3’UTR	28273	Extragenic	73.1		
23063A>T	S	N501Y	Missense	65.7	97.9	
25596A>T	ORF3a	R68S	Missense	20.4	0.1	
28095A>T	ORF8	K68*	Stop-gained	19.1	35.4
28250.>CTG	ORF8	L120	Insertion	14.8	0.04	
28254A>.	ORF8	F120	Deletion-frameshift	14.3	0.2	
1643A>T	NSP2	N280Y	Missense	13.9	2.4	
28271A>.	3’UTR	28271	Extragenic	13.9		
17615A>G	Helicase/NSP13	K460R	Missense	13.2	20.8	
2395C>T	NSP2	V530V	Silent	13.0		
3177C>T	NSP3	P153L	Missense	12.4	3.0	
28245T>G	ORF8	L118V	Missense	9.1	1.5	
29686C>G	3’UTR	29686	Extragenic	7.8		
8603T>C	NSP4	F17L	Missense	5.4	4.0	
15096T>C	NSP12b	N543N	Silent	4.3		
12162A>G	NSP8	Q24R	Missense	4.1	6.2	
2453C>T	NSP2	L550F	Missense	4.1	7.7	
8590A>G	NSP4	K12K	Silent	3.5		
25252G>T	S	V1230V	Silent	3.3		
25437G>T	ORF3a	L15F	Missense	2.8	3.7	
12970C>T	NSP9	N95N	Silent	2.8		
23012G>A	S	E484K	Missense	2.6	0.3	
8179G>A	NSP3	R1820R	Silent	2.4		
29109C>A	N	P279Q	Missense	2.4	0.3	
26730G>C	M	V70L	Missense	2.4	1.1	
3096C>T	NSP3	S126L	Missense	2.2	0.4	
4255G>A	NSP3	P512P	Silent	2.2		
16391C>T	NSP13	A52V	Missense	2.2	0	
16726C>T	NSP13	H164Y	Missense	2.2	0	
29272C>T	N	Y333Y	Silent	2.2		
21843C>T	S	S94F	Missense	2.0	0.4	
12933C>T	NSP9	P83L	Missense	2.0	0	
7272G>A	NSP3	R1518K	Missense	2.0	0	
8290C>T	NSP3	L1857L	Silent	2.0		
19164C>T	NSP14	D375D	Silent	2.0		

Interestingly, Y145 deletion in Spike protein was found to have 95.7% prevalence in Pakistan but globally this mutation had a prevalence of just 4.2%. Likewise, R68S substitution mutation in ORF3a; L120 insertion, F120 frameshift deletion, L118V substitution in ORF8 protein; N280Y in NSP2; and P153L in NSP3 protein had 20.4%, 14.8%, 14.3%, 9.1%, 13.9% and 12.4% prevalence locally but globally they had just 0.1%, 0.2%, 0.04%, 1.5%, 2.4%, and 3% of prevalence respectively (Table 4). On the other hand, N501Y (Spike) and K460R (NSP13) substitution mutations had 97.9% and 20.8% prevalence globally but, in our samples, their frequencies were decreased to 65.7% and 13.2% respectively.

### Phylogenetic analysis

**Fig 3:**
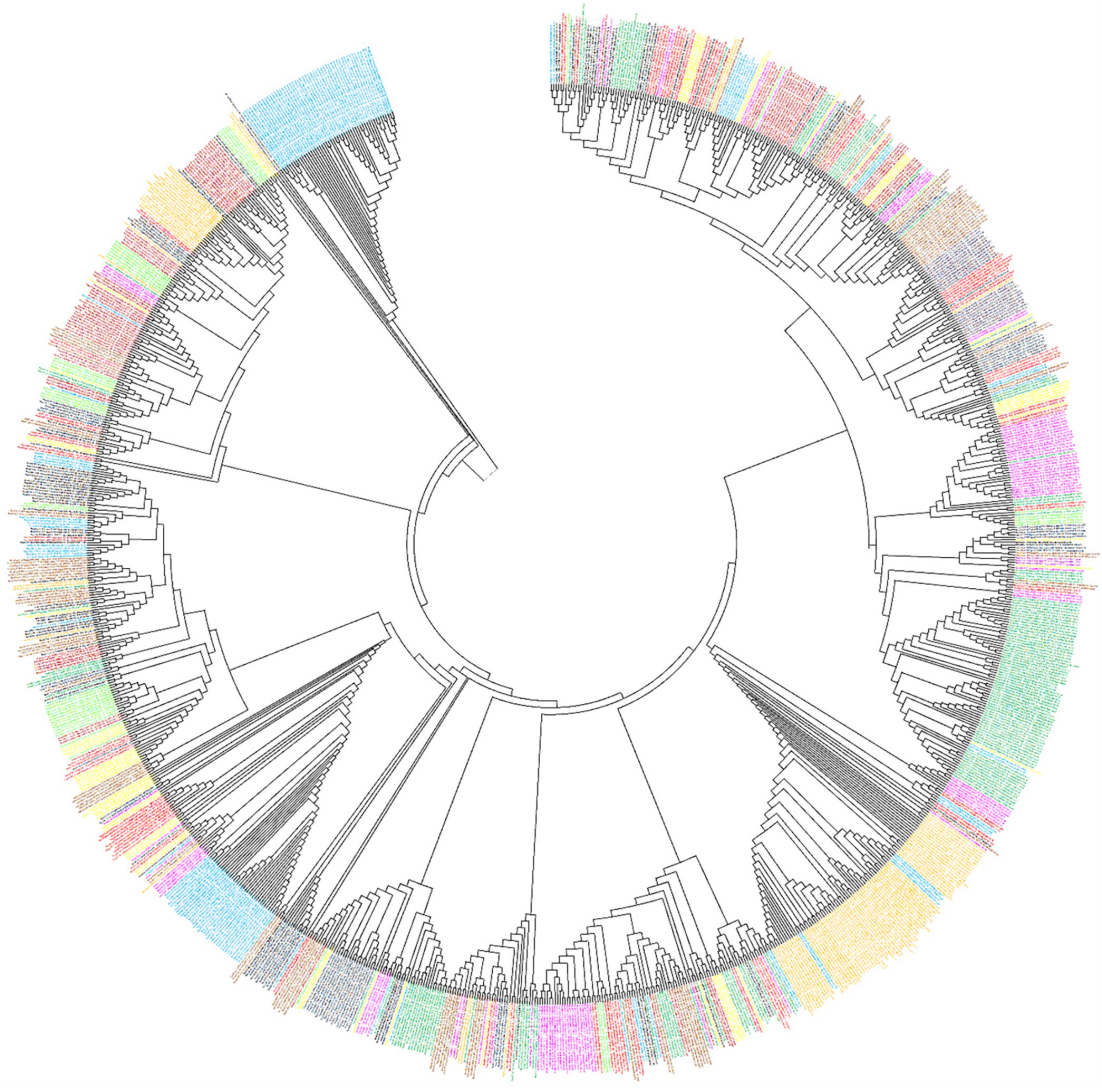
Maximum-likelihood based phylogenetic tree of 1300 samples of alpha-variant genomes of SARS-CoV-2 reported from the Pakistan and from the countries where most of the Pakistani diaspora is residing (Australia, Canada, England, Oman, France, Germany, Italy, Saudi Arabia, South Africa, Spain, UAE, USA, Northern Ireland, and Pakistan).

Phylogenetic tree was constructed by using the 1100 whole-genome sequences of B.1.1.7 variant from the countries (100/country), where most of the Pakistani diaspora is residing and used to travel to Pakistan very frequently. The results of phylogenetic analysis (Fig 3) revealed that major cluster of 78 Pakistani samples showed close relationship with samples originated from Italy. It was followed by the grouping of our 10 and 15 other samples with those of Spain in two separate clusters. And many of our other samples were grouped together with the samples reported from England, France, Scotland, Wales, and Northern Ireland in some smaller clusters. However, surprisingly our data did not reveal any relationship with the samples reported from UAE and Saudi Arabia though a substantial number of Pakistani diasporas reside there.

## Discussion

In this study, we identified a diverse array of genomic variations in the population of alpha-VOC of circulated in Pakistan and, interestingly, some of these mutations have very different frequencies in the Pakistani and global population of this variant. In total, we detected 1725 mutations which were further categorized into 899 amino acids changing mutations, 654 silent mutations, 52 mutations in non-coding regions, 25 in-frame deletions, 51 frameshift deletions, 01 in-frame insertions, 21 frameshift insertions, 21 stop-gained variants, and 1 deletion-stop variant (Table 3). In consistent with our findings many other authors have reported an ongoing divergence in the genome of SARS-CoV-2, owing to its continuous and rapid evolution, compared with its reference genome (Wuhan-Hu-1) [12, 21–26]. These findings are further corroborated with the emergence of many new variants of this virus, accumulating a large number of mutations which have enhanced their fitness, transmission and pathogenicity [27–30]. Out of the 899 missense variants, 537 were found in ORF1ab, which transcribe into 16 nonstructural proteins (NSPs). However, among all the nonstructural proteins, the NSP3 protein which is involved in viral replication [31] had the largest number of mutations (355), followed by NSP2 which had the second highest number of mutations (146) among all the NSPs. In agreement with our results Koyama et al. [25] also detected the largest number of mutations in NSP3, followed by NSP2 among all the nonstructural proteins in their analysis of 10,022 genomes of SARS-CoV-2. Out of the 22 characteristic mutations of alpha-VOC, 17 mutations including H69 (Δ69), A570D, P681H, D614G, T716I, S982A, D1118H in Spike protein; P314L in NSP12b protein; T183I, A890D, I1412T in NSP3; D3L, RG203KR, S235F in N; Q27*, Y73C, and R521 in ORF8 were also present in Pakistani samples with a prevalence of 95–100% (Table 4). However, N501Y,–a major characteristic mutation of alpha-VOC–present in the receptor-binding domain (RBD) of spike protein which bind with human ACE2 enzyme had a global prevalence of 97.9% but in Pakistan, it was just 65.7% prevalent. The mutations in RBD region are reported to affect the antibody recognition and a 5–10 times increase in the ACE2 binding affinity [1, 32, 33], which subsequently enhance viral transmissibility, contagiousness, and infectivity [34]. Hence, the decrease in the prevalence of this mutation indicate that Pakistani population of alpha-variant was comparatively less virulent compared with the global populations. However, surprisingly some other characteristic mutations like Δ144(S), G204R(N), and T1001I, I2230T, del3675–3677 (ORF1ab) which are also known as characteristic mutations of this variant were not present in the Pakistani population though globally, they had >90% prevalence. Likewise, Y145 deletion (Δ145) in the N-terminal domain (NTD) of spike protein had a global prevalence of 4.2% only but in Pakistan it was 95.9% prevalent; however, another characteristic mutation–Y144 deletion (Δ144)- in the NTD region was not present in our samples. The amino acid Y144, Y145, and V146 configure a conservative pocket in the NTD region of the S1 subunit of spike protein and deletion of any of these residues can result in changing the affinity between NTD and endogenous mAbs and the disruption of cell entry [35, 36]. Hence, the absence of Δ144 and a very high frequency of Y145 deletion in our samples could also be a reason for the low fitness, and less virulence of alpha-variant in Pakistan compared with its global population. And it could be the reason that the 3^rd^ wave of SARS-CoV-2 which had the highest (72.7%) prevalence of alpha-variant in the country could only cause 335,728 infections and 7,849 deaths [16]. However, on the other hand, in USA, UK and other European countries this variant caused millions of infections in each of these countries [37–39].

Among the accessory proteins, the ORF3a had the largest number of mutations (95) followed by ORF8(56), ORF7a(53), ORF10(13), ORF7b(11), and ORF6(10) (Table 3). The ORF3a is a highly conserved and the largest accessory protein of SARS-CoV-2, which is involved in virus release, apoptosis and pathogenesis [40, 41]. In this study, a substitution mutation (R68S) was identified in this protein with a prevalence of 20%, however, globally its prevalence was just 0.1%. Likewise, in case of ORF8, four signature mutation including K68*, F120 frameshift deletion, L120 frameshift insertion, and L118V substitution with a prevalence of 19.1%, 14.3%, 14.8%, and 9.1% were found, whereas globally they were 35.4%, 0.2%, 0.04%, and 1.5% prevalent respectively (Table 4). The K68* -a stop-gained mutation in ORF8- was identified with a low frequency by the end of December 2020, but its frequency was rapidly increased to 35.4% in the first few months of 2021 [42], however, in Pakistan frequency of this mutation was only 19.1% which is nearly half of its global prevalence. These mutations in ORF8 of the alpha-variant of SARS-CoV-2 were also observed by some other authors [43] and were reported to be likely involved in immune evasion and cytokine response mimicking [43]. The characterization of mutations, in ORF8 protein is important not only for pathogenesis and immune modulation but also for the drugs and diagnostic tests, as this viral protein has been shown to elicit strong and specific antibody response [44, 45]. In case of NSPs, N280Y, L550F in NSP2 and P153L, S126L and R1518K in NSP3 were some emerging mutations with a prevalence of 13.9%, 4.1%, 12.4%, 2.2% and 2.0% in Pakistan but globally they were only 2.4%, & 7.7%, 3.0%, 0.4% and 0.0% prevalent respectively (Table 4). In agreement with our findings Koyama et al. [25] also found P153L as the most common mutation in NSP3, though its frequency was only 0.01%. Both NSP2 and NSP3 are reported to be involved in the formation of transcription and replication complexes and enhancing the half-lives and functioning of other proteins in the cytoplasm [40], hence, it is highly likely that these mutations have their roles in the transcription and replication processes of the virus. However, the exact role of these mutations remains to be determined and merit further investigation.

In the helicase protein, we also detected some missense mutations such as A52V, H164Y, and K460R with a frequency of 2.2%, 2.2%, and 13.2% respectively. The global prevalence of K460R mutation was 20.8% [46], however, the other two variants (A52V and H164Y) were not reported earlier due to which their global prevalence was not known. Likewise, in RdRp/NSP12b T903T, P314L, P403P, H604H were detected which had 98–100% prevalence locally and globally. The P314L mutation is located very closely to the drug binding region in the hydrophobic cleft of RdRp which is the target of some antiviral drugs like remdesivir and favipiravir [47, 48]. Occurrences of highly prevalent mutations in this protein suggest that some therapeutic resistance strains of this virus are likely to emerge very shortly. In case of untranslated region, we detected 13 mutations in the 5’UTR and 39 in the 3’UTR of alpha-variant genome and among all of these mutations, C>T variation at 241bp in the 5**´**UTR, 28273bp, and 28271bp in 3’UTR appeared most predominantly with a frequency of 78.3%, 73.1% and 13.9% respectively. Mutations in the 5’UTR and 3’UTR region can have a significant impact on folding, transcription and replication of the viral genome [47]. In agreement with our results, 241C>T substitution in 5’UTR has been reported as a frequent mutation globally [18] which is involved in increasing the binding of Trans-active Response DNA binding protein (TARDBP) to 5’UTR of SARS-CoV-2 genome which enhance the multiplicative ability of the virus within the host [49]. Additionally, some missense mutations like R1518K (NSP3), P83L (NSP9), and A52V, H164Y (NSP13) were found for the very first time in our population of alpha-VOC which were not reported earlier (Table 4). Hence, the exact role of these mutations is not known and merit further investigations.

The results of phylogenetic analysis showed that a set of 78 Pakistani samples of alpha-VOC was clustered together and showed close genetic relationship with the variant reported from Italy. However, in two other separate clusters, 10 and 15 Pakistani samples were grouped together with the samples reported from Spain exhibiting close association to those samples (Fig 3). Though the alpha-variant was originated from UK–a country which inhabit the second largest diaspora (1.2 million) of Pakistani peoples which are used to travel to their homeland frequently. But surprisingly, the major cluster of Pakistani samples of B.1.1.7 showed closed proximity with the Italian samples, though Italy is the eighth major country for inhabiting the Pakistani diaspora. However, in addition to these countries, remaining scattered samples were grouped with those reported England, South Africa, France, Scotland, USA, Wales, and Northern Ireland. Surprisingly our data did not reveal any relationship of our samples with those reported from Saudi Arabia and UAE though a substantial number of Pakistani diasporas reside in these two countries. Taken together, the results of phylogenetic analysis suggest that alpha-variant was mainly transmitted to Pakistan from the western Europe.

## Conclusions

In this study, we identified 1725 mutations in the genome of alpha-variant population of SARS-CoV-2 circulated in Pakistan. The NSP3 and Spike protein were found as the most variable protein with 356 and 223 mutations respectively. Out of the 22 characteristic mutations of alpha-VOC, 16 were present with 95–100% prevalence, whereas some other characteristic mutations like Δ144(S), G204R(N), T1001I, I2230T, and del3675–3677 (ORF1ab) were missing in the Pakistani population of alpha-VOC. Some new missense mutations like A52V, H164Y (NSP13), R1518K (NSP3), and P83L (NSP9) were found for the very first time in our population of alpha-VOC of SARS-CoV-2 and they were not reported earlier. Likewise, N501Y(Spike) and K460R(NSP13) substitution mutations had 97.9% and 20.8% prevalence globally but, in Pakistan, their frequencies were decreased to 65.7% and 13.2% respectively. Interestingly, Y145 deletion in Spike protein was found to have 95.7% prevalence in Pakistan but globally this mutation was just 4.2% prevalent. Likewise, R68S substitution mutation in ORF3a, L120 insertion, F120 frameshift deletion, L118V substitution in ORF8, N280Y in NSP2 and P153L in NSP3 protein had prevalence of 20.4%, 14.8%, 14.3%, 9.1%, 13.9% and 12.4% locally but globally they were just 0.1%, 0.2%, 0.04%, 1.5%, 2.4%, and 3% prevalent respectively. We hereby recommend to continue and enhance the level of genomic surveillance in this pandemic in order to develop some genome-based diagnostics, and biologicals (vaccines or therapeutics) for the prevention and treatment of COVID-19.

## Supporting information

S1 Fig (a) Health status, (b) gender of the patient affected by Alpha variant of SARS-CoV-2 in Pakistan.
(TIF)

S2 Fig Age group of patients affected by alpha variant of SARS-CoV-2 in Pakistan. (TIF)

S1 Data (XLSX)
