# Supplementary figures and images for: Genomic landscape of alpha-variant of SARS-CoV-2 circulated in Pakistan
Source: PLoS One. 2022 Dec 13;17(12):e0276171. doi: 10.1371/journal.pone.0276171 (PMC9746927; doi:10.1371/journal.pone.0276171)

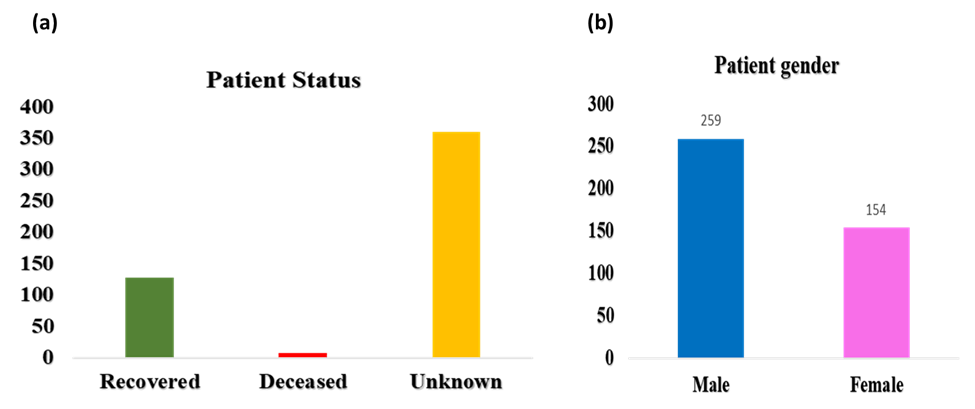

Supplement: S1 Fig — (a) Health status, (b) gender of the patient affected by Alpha variant of SARS-CoV-2 in Pakistan. (TIF) [file pone.0276171.s001.tif]

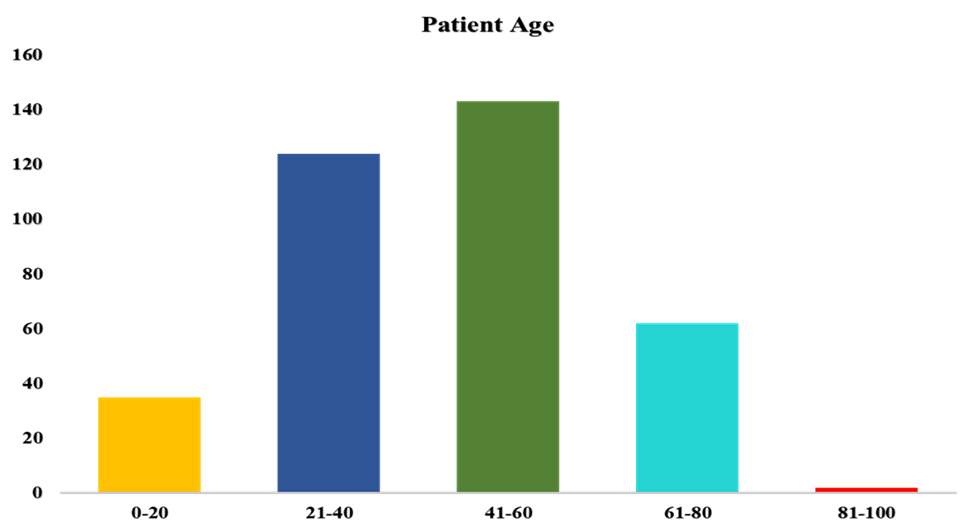

Supplement: S2 Fig — (TIF) [file pone.0276171.s002.tif]
